# The Therapeutic Value of Veratrum Viride

**Published:** 1853-06

**Authors:** W. C. Norwood

**Affiliations:** Abbeville, South Carolina


*The Therapeutic value of Veratrum Viride.* By W. C. Norwood, M. D., of Abbeville, South Carolina.—We know of no agent so peculiarly adapted and so universally applicable to the treatment of febrile and inflammatory affections, as the American Hellebore. With limited exceptions it fulfils the therapeutical indications in these diseases unaided and alone; and, withal, its results are so invariable that we may expect to realize its effects, when properly administered, with the same certainty with which the husbandman anticipates an abundant harvest from the proper culture of his field.

However numerous or diversified may be the causes of febrile and inflammatory diseases, certain uniform effects or events are sure to follow them, and to correct or modify these effects is the grand aim of the practitoner. To do this successfully, a practical and experimental knowledge of the therapeutical properties of the agents calculated to modify the actions excited by these causes, is necessary; and this is the reason of my pressing so earnestly on every one, the importance of thoroughly testing, and closely watching for himself, the therapeutical effects of veratrum viride; for I believe that he who fully understands its powers and applications, is in possession of the key which opens the door to triumphant success in the treatment of many diseases hitherto considered the opprobria of medicine.

I have been charged with enthusiasm, extravigance, and fancy, in estimating the value of this remedy, and in urging its claims upon the profession; but years of close observation and experience have convinced me that, far from exaggerating, I have failed to do justice to the subject. Who can overrate the value of an agent capable of controlling the actions of the heart with demonstrative certainty? When it was announced, by the Italian physicians, that digitalis possessed the property of subduing and controlling morbid vascular action, medical men ardently anticipated the benefits that were about to be realized. But it proved not to possess the powers ascribed to it, and the field was again open for research and inquiry. After long observation and repeated experiment we are able to affirm, in the most positive manner, that *veratrum viride* entirely fulfils this indication. In less than twenty-four hours it will reduce a pulse of one hundred, or one hundred and sixty, to between thirty-five and eighty-five beats in the minute.

But this drug possess s other important remedial powers. It is a certain and efficacious emetic; it possesses expectorant and diaphoretic properties in an eminent degree: its adanagic power is considerable, and it acts as a nervine in allaying morbid irritability.

We trust that these statements, based upon careful clinical observation and mature reflection, will induce the profession to use this remedy, and we believe that the benefits resulting to the sick from its judicions employment will be incalculable.*—*Virginia Medical and Surgical Journal.*

*Dr. Norwood publishes (*Charleston Medical Journal*, November, 1852,) his formula for preparing a saturated tincture of the root of veratrum viride:—℞· Of the dried root of veratrum viride, ℥viij; of alcohol of the shops, ℥xvj. Digest for a fortnight. The dose for an adult is eight drops, to be given every three hours.—[Ed. V. M. J.

